# Primary bone lymphoma of the mandible and thyroid incidentaloma identified by ^18^FDG PET/CT: a case report

**DOI:** 10.4076/1757-1626-2-6384

**Published:** 2009-06-26

**Authors:** Joaquim Bosch-Barrera, Leire Arbea, Maria J García-Velloso, Ignacio Gil-Bazo, Jesús García-Foncillas, Carlos Panizo

**Affiliations:** 1 Department of Oncology, Clínica Universidad de NavarraPamplonaSpain; 2 Department of Nuclear Medicine, Clínica Universidad de NavarraPamplonaSpain; 3 Department of Hematology, Clínica Universidad de NavarraPamplonaSpain

## Abstract

The mandible is a rare site for the occurrence of primary bone lymphoma (PBL), a non-Hodgkin lymphoma. We report herein a case of an incidentally diagnosed thyroid incidentaloma by ^18^Fluorodeoxyglucose Positron Emission Tomography/Computed Tomography in a patient with a previous diagnosis of PBL. Therapeutic options are reviewed and discussed.

## Introduction

Primary bone lymphoma (PBL) represents approximately 3% of all malignant bone tumors [1] and 5% of all non-Hodgkin lymphomas. Most occurrences present similar characteristics such as a slightly greater frequency in men, early-stage disease presentation and a median age of appearance in the fifth or sixth decade. PBL can appear in any part of the skeleton. The most common tumor sites are the long bones (50% extremities), followed by the axial skeleton (44%). The mandible is a rare location, comprising approximately only 2-4% of all PBLs [2,3].

Establishment of useful diagnostic and therapeutic guidelines is problematic because of the infrequency of PBLs.

PBL therapy has evolved from radiotherapy as a standalone strategy (since the 1960s) to combined modality therapy (chemotherapy and radiotherapy) [4]. There have also been a few reports of patients treated with chemotherapy alone [2,3]. Surgery is currently limited to diagnostic procedures and repair of pathological fractures of the bone.

Diffuse large B-cell lymphoma (DLBCL) is the most frequent histopathological subtype of PBL. Standard chemotherapy for patients with DLBCL consists of administration of CHOP (cyclophosphamide, doxorubicin, vincristine, and prednisone) in conjunction with the monoclonal antiCD20 rituximab [5-7]. Treatment options differ for patients with localized (Ann Arbor stage I-II) or advanced (Ann Arbor stage III-IV) disease. We present the case of a PBL that affected the mandible. Therapeutic options are reviewed and discussed.

## Case presentation

**Figure 1.**
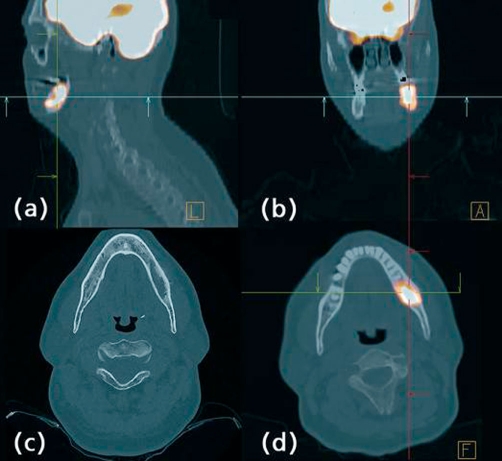
^18^FDG PET/CT showing the left mandible lesion in sagital **(a)**, coronal **(b)** and axial **(d)** view. Image **(c)** shows an osteolytic lesion in the left mandible in an axial view of the CT scan.

A 47-year-old Caucasian Spanish man presented with a history of eight months of dental pain. Despite extraction of two teeth (numbers 37 and 38), improvement of symptoms was not achieved. A computed tomography (CT) scan of the head revealed an osteolytic lesion on the left mandible of 3.8 × 1 cm (Figure 1). A CT-guided biopsy was performed. Histopathological examination of the biopsy revealed a DLBCL; immunohistochemistry analysis tested positive for cytoplasmic CD20, Bcl-2 and nuclear Bcl-6.

**Figure 2.**
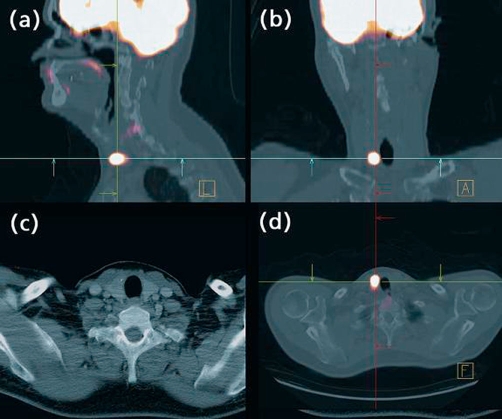
^18^FDG PET/CT showing the thyroid incidentaloma in sagital **(a)**, coronal **(b)** and axial **(d)** view. Image **(c)** shows the right thyroid cyst on the CT scan.

The disease was staged using a bone marrow biopsy, CT body scan and fluorodeoxyglucose positron emission tomography (FDG PET). CT scan revealed a lesion on the left mandible as well as the presence of a cyst on the right thyroid. ^18^FDG PET/CT showed high uptake of tracer in the left mandible lesion with a standard uptake value (SUV) of 5.5 (Figure 1) and a higher focal uptake in the right thyroid, with an SUV of 8 (Figure 2). The bone marrow biopsy and hematological and chemical analysis were normal. A fine-needle aspiration (FNA) guided by echography of the thyroid lesion revealed a papillary thyroid carcinoma.

The final diagnosis was DLBCL stage I EA with a low IPI score and a synchronic papillary thyroid carcinoma.

The papillary thyroid carcinoma was treated first with a total thyroidectomy. Anatomopathological examination revealed a follicular variant of papillary carcinoma of the thyroid of 1.5 cm with pT1pN1aM0 stage (stage III). The patient began treatment with levothyroxine.

The lymphoma was treated with six cycles of chemotherapy (R-CHOP) with good tolerance. An ^18^FDG PET/CT was performed at the end of the treatment with no pathological signal.

After chemotherapy the patient was irradiated with 3D conformational radiotherapy. The left mandible branch received 30 Gy in 20 fractions of 2.0 Gy followed by 6 Gy as a boost to the initial tumor volume.

One month later, the patient received adjuvant radioiodine ablation with I^131^ for the thyroid carcinoma. The patient has been completely tumor-free of the two tumors for 28 months, with the last follow-up in January 2009.

## Discussion

Presentation symptoms of PBL include localized bone pain and sometimes the presence of a palpable mass. In the mandible, the main symptoms of PBL are pain, swelling, numbness, tooth mobility and cervical lymphadenopathy. In this case, the patient had what appeared to be dental caries, which were previously evaluated by a dentist. Initial diagnosis is often wrong; Limb et al. suggested that the mean time to achieve a correct diagnosis of PBL is 8 months [1].

The role of FDG-PET in the management of patients with lymphoma is clear. It has the potential to influence both the initial choice of chemotherapy and alterations to the management of the disease based on the response to therapy [8]. In our case, the ^18^FDG PET/CT provided staging information of the lymphoma and revealed the presence of a hypermetabolic lesion on the thyroid that was finally diagnosed as papillary carcinoma.

Thyroid incidentalomas are defined as newly identified thyroid lesions encountered during imaging studies. As ^18^FDG PET/CT is becoming a more common imaging modality, the incidence of thyroid incidentalomas is also increasing. A few retrospective studies have shown the prevalence of this finding to be approximately 2.3% [9]. Kang et al. studied the possibility of differentiating a malignant from a benign lesion with SUV. While a SUV > 9 suggested a higher likelihood of cancer, the authors concluded that a pathological confirmation is required.

The value of FDG-PET in patients with differentiated thyroid cancer is under review. FDG-PET has a clear role in I^131^-negative thyroid cancer and follow-up, but it should not be performed in patients with a stimulated Tg < 10 μgr/L because of its low sensitivity [10,11].

The treatment and optimal management of patients with PBL are unclear because of the low incidence of this pathology. Few retrospective studies have examined the clinical characteristics, treatment and outcome of these patients. The published studies span many years, during which time staging techniques and treatment modalities have changed. Despite the heterogeneity of the treatment in the literature, it seems clear that a combined modality therapy of chemotherapy with radiotherapy leads to better results than a single modality of treatment [2,12]. Someya et al. reviewed the literature regarding the treatment of PBL of the mandible and concluded that radiotherapy alone was not enough for tumor control for stage I + II disease and that combination chemotherapy might be needed [13]. This conclusion is consistent with the results of studies on patients with early-stage non-Hodgkin lymphoma [14,15].

Rituximab added to six cycles of CHOP is an effective treatment for young patients with good-prognosis DLBCL [7] and is currently the gold standard treatment for these patients. This regimen results in improvement of both event-free survival and survival. The excellent results achieved in patients with stage I without bulky disease suggest that the addition of rituximab may lead to a reduction in chemotherapy cycles and may therefore be an option for the future treatment of these patients.

## Abbreviations

CT: Computed tomography
DLBCL: Diffuse large B-cell lymphoma
FDG PET: fluorodeoxyglucose positron emission tomography
FNA: Fine needle aspiration
PBL: Primary bone lymphoma
SUV: Standard uptake value

## References

[bib-001] Limb D, Dreghorn C, Murphy JK, Mannion R (1994). Primary lymphoma of bone. Int Orthop.

[bib-002] Beal K, Allen L, Yahalom J (2006). Primary bone lymphoma: treatment results and prognostic factors with long-term follow-up of 82 patients. Cancer.

[bib-003] Zinzani PL, Carrillo G, Ascani S, Barbieri E, Tani M, Paulli M (2003). Primary bone lymphoma: experience with 52 patients. Haematologica.

[bib-004] Fidias P, Spiro I, Sobczak ML, Nielsen GP, Ruffolo EF, Mankin H (1999). Long-term results of combined modality therapy in primary bone lymphomas. Int J Radiat Oncol Biol Phys.

[bib-005] Coiffier B (2002). Rituximab in the treatment of diffuse large B-cell lymphomas. Semin Oncol.

[bib-006] Feugier P, Van Hoof A, Sebban C, Solal-Celigny P, Bouabdallah R, Ferme C (2005). Long-term results of the R-CHOP study in the treatment of elderly patients with diffuse large B-cell lymphoma: a study by the Groupe d'Etude des Lymphomes de l'Adulte. J Clin Oncol.

[bib-007] Pfreundschuh M, Trumper L, Osterborg A, Pettengell R, Trneny M, Imrie K (2006). CHOP-like chemotherapy plus rituximab versus CHOP-like chemotherapy alone in young patients with good-prognosis diffuse large-B-cell lymphoma: a randomised controlled trial by the MabThera International Trial (MInT) Group. Lancet Oncol.

[bib-008] Jhanwar YS, Straus DJ (2006). The role of PET in lymphoma. J Nucl Med.

[bib-009] Kang KW, Kim SK, Kang HS, Lee ES, Sim JS, Lee IG (2003). Prevalence and risk of cancer of focal thyroid incidentaloma identified by 18F-fluorodeoxyglucose positron emission tomography for metastasis evaluation and cancer screening in healthy subjects. J Clin Endocrinol Metab.

[bib-010] Palmedo H, Bucerius J, Joe A, Strunk H, Hortling N, Meyka S (2006). Integrated PET/CT in differentiated thyroid cancer: diagnostic accuracy and impact on patient management. J Nucl Med.

[bib-011] Stokkel MP, Duchateau CS, Dragoiescu C (2006). The value of FDG-PET in the follow-up of differentiated thyroid cancer: a review of the literature. Q J Nucl Med Mol Imaging.

[bib-012] Rathmell AJ, Gospodarowicz MK, Sutcliffe SB, Clark RM (1992). Localised lymphoma of bone: prognostic factors and treatment recommendations. The Princess Margaret Hospital Lymphoma Group. Br J Cancer.

[bib-013] Someya M, Sakata K, Nagakura H, Itou K, Nakata K, Oouchi A (2005). Three cases of diffuse large B-cell lymphoma of the mandible treated with radiotherapy and chemotherapy. Radiat Med.

[bib-014] Miller TP, Dahlberg S, Cassady JR, Adelstein DJ, Spier CM, Grogan TM (1998). Chemotherapy alone compared with chemotherapy plus radiotherapy for localized intermediate- and high-grade non-Hodgkin's lymphoma. N Engl J Med.

[bib-015] Yahalom J, Varsos G, Fuks Z, Myers J, Clarkson BD, Straus DJ (1993). Adjuvant cyclophosphamide, doxorubicin, vincristine, and prednisone chemotherapy after radiation therapy in stage I low-grade and intermediate-grade non-Hodgkin lymphoma. Results of a prospective randomized study. Cancer.

